# An Update on the Use of Animal Models in Diabetic Nephropathy Research

**DOI:** 10.1007/s11892-015-0706-2

**Published:** 2016-01-27

**Authors:** Boris Betz, Bryan R. Conway

**Affiliations:** Centre for Inflammation Research, University of Edinburgh, Edinburgh, Scotland; Centre for Cardiovascular Science, Queen’s Medical Research Centre, University of Edinburgh, 47 Little France Crescent, Edinburgh, EH16 4TJ Scotland; Department of Clinical Chemistry and Laboratory Medicine, Jena University Hospital, Jena, Germany

**Keywords:** Diabetic nephropathy, Animal models, Hypertension, Transcriptomics, Regression

## Abstract

In the current review, we discuss limitations and recent advances in animal models of diabetic nephropathy (DN). As in human disease, genetic factors may determine disease severity with the murine FVB and DBA/2J strains being more susceptible to DN than C57BL/6J mice. On the black and tan, brachyuric (BTBR) background, leptin deficient (ob/ob) mice develop many of the pathological features of human DN. Hypertension synergises with hyperglycemia to promote nephropathy in rodents. Moderately hypertensive endothelial nitric oxide synthase (eNOS^−/−^) deficient diabetic mice develop hyaline arteriosclerosis and nodular glomerulosclerosis and induction of renin-dependent hypertension in diabetic Cyp1a1mRen2 rats mimics moderately severe human DN. In addition, diabetic eNOS^−/−^ mice and Cyp1a1mRen2 rats recapitulate many of the molecular pathways activated in the human diabetic kidney. However, no model exhibits all the features of human DN; therefore, researchers should consider biochemical, pathological, and transcriptomic data in selecting the most appropriate model to study their molecules and pathways of interest.

## Introduction

Although there are tentative signs that the incidence of end-stage kidney disease due to diabetes is stabilizing, diabetic nephropathy (DN) remains the most common cause of end-stage renal disease in the western world [1]. Improvements in the management of hyperglycemia and hypertension have reduced the proportion of patients with diabetes reaching end-stage kidney disease [2]; however, additional therapies are required to target those with progressive renal disease. Unfortunately, the results of recent clinical trials in DN have been largely disappointing [3–5], and no new therapies that specifically target progression of nephropathy have been successfully translated into clinical practice in recent years [6].

## Role of Animal Models in Diabetic Nephropathy

One of the roadblocks in developing novel therapies for DN has been the lack of reliable preclinical models. For many diseases, rodent models have been useful in dissecting the pathogenesis of disease and for testing novel therapies. Indeed, the availability of genetically modified animals facilitates mechanistic studies that cannot be performed in humans. For example, by introducing genetic modifications, specific cell types can be fluorescently tagged to track the fate of the cells over time or to facilitate isolation of cells from whole organs, and this may enhance our knowledge of disease pathogenesis. In addition, targeted knockout or overexpression of genes can incisively determine the role of specific molecules in disease and whether such agents represent novel therapeutic candidates. However, the utility of animal models in DN research has been constrained by the fact that most models fail to recapitulate important functional, structural, and molecular pathological features of advanced human diabetic kidney disease [7, 8]. This may account for the fact that many therapies which have been found to be of benefit in preclinical models have not proved effective in clinical trials.

In order to rationalize the development of novel preclinical models of DN, the nephropathy subcommittee of the Diabetic Complications Consortium (DiaComp) has published criteria that can be used to validate animal models of DN [9]. These are based on the clinical and pathological features of human DN and include the following: (i) a decline in renal function of more than 50 % over the lifespan of the animal; (ii) a greater than 10-fold increase in albuminuria; and (iii) key pathologic features of human disease including advanced mesangial matrix expansion (+/−nodules), thickening of the glomerular basement membrane, arteriolar hyalinosis, and tubulointerstitial fibrosis. Unfortunately, no currently available model meets all of these criteria [7].

## Animal Models of Diabetes

A number of animal models of both type 1 diabetes and type 2 diabetes are widely available to researchers (Table 1). Type 1 diabetes may be induced in rodents by administration of streptozotocin, which is toxic to β-cells, resulting in absolute insulin deficiency. Streptozotocin may have toxic effects on other organs including the kidney, although these may be minimized by administering smaller doses over five consecutive days. Genetic models of type 1 diabetes are also available, including Akita [10] and OVE26 [11] mice, in which mutations in the insulin and calmodulin genes, respectively, result in toxic accumulation of a defective protein specifically in pancreatic β-cells.

**Table 1:** Summary of commonly utilized animal models of diabetic kidney disease with brief description

Genetic modification and strain	Description	Ref.
Models of diabetes		
DBA/2J mice	T1DM, STZ injection induces diabetes on a strain susceptible for nephropathy	[18•]
Ove26 on FVB mice	T1DM, mutation in the calmodulin gene results in toxic accumulation of defective proteins in beta-cells (nephropathy can be further exacerbated by uninephrectomy)	[11, 20, 43]
Akita (Ins2+/C96Y) on C57BL6 mice	T1DM, mutation in the insulin gene results in misfolding and toxic accumulation of insulin	[10]
db/db mice (C57BLKS/FVB)	T2DM, inactivating mutation in the leptin receptor leading to hyperphagia and obesity	[19]
ob/ob on BTBR mice	T2DM, leptin deficiency on a black and tan, brachyuric strain that is naturally insulin resistant	[21••]
MKR on FVB mice	T2DM, dysfunctional insulin-like growth factor-1 receptor (IGF-1R) in the skeletal muscle results in nonobese insulin resistance. Nephropathy may be exacerbated by uninephrectomy or high-fat diet	[15]
fa/fa on Zucker rat	T2DM, hyperphagic and obese, due to missense mutation in the gene coding the leptin receptor	[14]
OLETF rat	T2DM, hyperphagia and obese, in part due to a spontaneous mutation in cholecystokinin receptor-1	[25]
Goto Kakizaki rat	T2DM, polygenic, nonobese model with deficient insulin production and insulin resistance. Nephropathy may be exacerbated by diet-induced hypertension	[46]
Specific genetic modifications to accelerate nephropathy		
ApoE^−/−^ on C57BL6 mice	T1DM, STZ-induced diabetes combined with hyperlipidemia due to lack of apolipoprotein E	[56]
eNOS^−/−^ on C57BL6 or db/db mice	Vascular dysfunction and hypertension induced by eNOS deficiency accelerate renal injury in either STZ-induced diabetes (T1DM) or when backcrossed to db/db mice (T2DM)	[52, 53]
TTRhRen on FVB mice	T1DM, hypertension induced by human renin overproduction combined with diabetes induced by STZ injection or OVE26 mutation	[48]
Cyp1a1mRen2 on Fisher rat	T1DM, severe hypertension induced by renin overproduction synergises with STZ-induced diabetes	[47]

Refer to the text for a detailed description and characterization of the models

*STZ* streptozotocin, *MKR* MCK-KR-hIGF-IR mice, *OLETF rat* Otsuka Long-Evans Tokushima Fatty rat, *TTRhRen* transgenic mice expressing active human renin in the liver

Models of type 2 diabetes typically utilize genetically obese rodents, which are either leptin deficient (e.g., ob/ob mice [12]) or have inactivating mutations in the leptin receptor (e.g., db/db mice [13], Zucker rats [14]). These animals exhibit hyperphagy, obesity, and insulin resistance and develop relative insulin deficiency and hyperglycemia in the first 8 weeks of life. The degree of hyperglycemia is dependent on the nature of the mutation and on the background strain of the animal. Typically, hyperglycemia is less severe in ob/ob mice and in leptin receptor-deficient mice on the C57BL/6J background, whereas db/db mice on the C57BLKS background develop fulminant diabetes and require exogenous insulin administration in order to maintain well-being beyond 24 weeks of age. Administration of a high-fat diet is useful for investigating mechanisms of insulin resistance; however, the animals rarely become overtly hyperglycemic. Furthermore, the high-fat diet per se may promote renal injury, and the animals do not exhibit classical features of human DN. An alternative nonobese model of type 2 diabetes employs MKR mice in which the insulin receptor is dysfunctional specifically in skeletal muscle, resulting in insulin resistance, marked hyperglycemia, and hyperlipidemia [15].

## Choice of Rodent Species and Strain

Mice breed rapidly, are relatively cheap to house, and have long been amenable to genetic manipulation, and hence, they are the most widely used species in preclinical research; however, they tend to be resistant to the development of DN. In patients with diabetes, multiple genetic factors influence the risk of developing nephropathy [16], some of which are now being elucidated by genome-wide association studies [17]. Similarly in mice, the susceptibility to nephropathy is influenced by the particular strain of mouse employed [7, 9]. The C57BL/6J mouse is the most common strain used in preclinical research, and hence, many genetic modifications are performed on this background. Unfortunately, this strain is relatively resistant to the development of DN [18•]; therefore, a lengthy and expensive breeding program may be required to backcross genetic mutations onto a more susceptible strain such FVB and DBA/2J mice. For example, when the leptin receptor mutation found in db/db mice is crossed to the FVB background, the resultant mice are more susceptible to nephropathy than equally obese and hyperglycemic C57BL/6J mice [19]. In addition, OVE26 mice on the FVB background exhibit nodular glomerulosclerosis and a greater than 10-fold increase in albuminuria by 6 months of age [20]. Diabetic DBA/2J mice develop more marked albuminuria than C57BL/6J mice, and additionally, they exhibit some pathological features of human disease, such as nodular glomerulosclerosis and arteriolar hyalinosis [18•].

More recently, it has been shown that the relatively uncommon black and tan, brachyuric (BTBR) mouse may be a potentially useful strain for modeling DN. BTBR mice are naturally insulin-resistant, and when the ob/ob mutation is placed on this strain, the mice exhibit sustained hyperglycemia from an early age, in contrast to ob/ob mice on the C57BL/6J background. Furthermore, ob/ob BTBR mice develop some pathological features of human DN including arteriolar hyalinosis, mesangial expansion, mesangiolysis, focal nodular glomerulosclerosis, and a reduction in podocyte number [21••]. Unfortunately, ob/ob BTBR mice are difficult to breed and they have high mortality rates beyond 24 weeks of age, which limits their use in modeling more advanced nephropathy.

In all of these strains of mice, the increase in albuminuria is equivalent to modestly elevated levels in humans (typically ∼10-fold) and they do not develop a progressive decline in renal function. Furthermore, they exhibit at most mild tubulointerstitial fibrosis, which is important as this is the best pathological determinant of progressive DN in humans [22].

Prior to the advent of genetic modification of mice in the 1980s, the rat was the most commonly studied model organism as there are a number of advantages in using rats rather than mice to model disease [23]. Their greater size facilitates repeated blood sampling, monitoring of renal physiology, and access to sufficient renal tissue for analysis. Furthermore, rats are more susceptible than mice to many cardiovascular diseases including hypertension, and for many traits, the genetics and pathophysiology in rats has proven more relevant to human disease. Models of type 1 diabetes (streptozotocin-induced) and type 2 diabetes (Zucker, Goto Kakizaki [24], and Otsuka Long-Evans Tokushima Fatty (OLETF [25]) rats have been employed; however, these models typically do not develop features of advanced human DN. Recent advances have enabled targeted gene knockout in rats [26]; however, it will be several years before the high-throughput murine gene knockout programs can be recapitulated in rats and in the interim researchers will have to incur the additional cost of generating custom-made transgenic rodents.

Large animal species, such as pigs and dogs, have also been utilized to model DN. Following induction of type 1 diabetes by administration of alloxan, dogs develop glomerular lesions within 2 years [27] and this process may be accelerated by performing uninephrectomy [28]. Similarly, pigs have been used to model the early glomerular lesions of human DN and test therapeutic agents [29]. However, in these large animal models, there is no evidence of more advanced features of human DN, such as tubulointerstitial fibrosis or a decline in renal function. As the large animal models have few major advantages compared with rodent models, given the greater husbandry costs and prolonged study duration, it is likely that rodents will remain the preeminent species in preclinical DN research.

Model organisms such as *Drosophila* and zebrafish afford several advantages over rodents such as high fecundity, short lifespan, low breeding costs, ease of genetic manipulation, and relative ease of in vivo imaging of deep tissues. For these reasons, they have long been used to study fundamental processes such as development, apoptosis, and regeneration. Zebrafish can be rendered diabetic by repeated doses of streptozotocin [30] and have been used to study diabetes complications [31]. *Drosophila* possess nephrocytes, which exhibit features of both podocytes including slit diaphragms [32••], and proximal tubular cells including cubilin-mediated transport [33]. Administration of a high sucrose diet to *Drosophila* promotes nephrocyte dysfunction and induces changes in gene expression that mimic human DN [34]. However, the primitive renal cells in both zebrafish and *Drosophila* are clearly functionally very different to humans, and therefore, these models are most likely to be used in genetic or drug screens and the results will need replicated in mammalian preclinical models.

## Role of Hemodynamic Factors

The importance of hemodynamic factors in the pathogenesis of DN has long been recognized [35]. Patients with advanced DN invariably have hypertension and tight control of blood pressure is at least as important as glycemic control in slowing disease progression [36]. Hypertension may not simply be a consequence of nephropathy but may promote the development of kidney disease in diabetic patients. Subtle abnormalities in blood pressure, such as loss of nocturnal dipping, precede the onset of albuminuria [37] and inheritance of genetic variants that confer risk of hypertension promotes nephropathy in patients with diabetes [38]. In fact, hypertension may be an absolute requirement for progression of DN, as illustrated by two remarkable case reports. In both cases, the patients had long-standing diabetes and coexisting unilateral renal artery stenosis; they exhibited no evidence of nephropathy in the kidney downstream of the arterial stenosis, despite severe nephropathy in the contralateral kidney [39, 40].

Researchers have attempted to replicate these hemodynamic factors in rodent models in a number of ways. In seminal studies in the 1980s, a high protein diet was found to increase glomerular pressure and injury in diabetic rats [41], and the role of ACE inhibitors in slowing progression of DN was first proposed [42]. It is worth noting that these studies focusing on hemodynamic factors are among the few to have been successfully translated into clinical practice. An alternative method of applying hemodynamic stress that can be readily applied in most rodent models of DN is uninephrectomy. For example, unilateral nephrectomy in diabetic OVE26 mice accelerates many features of DN including albuminuria, inflammatory cell infiltration, fibrosis, and changes in gene expression [43]. However, caution should be applied to the results from these studies as the abnormal glomerular hemodynamics induced by uninephrectomy may not be representative of the pathophysiology of human DN.

It has been consistently demonstrated in rodent models that the combination of diabetes and genetic hypertension results in more severe albuminuria, glomerulosclerosis, and tubulointerstitial fibrosis than diabetes alone [44–46, 47•]. Given the importance of the renin-angiotensin-aldosterone system (RAAS) in human DN, several researchers have employed transgenic rodents in which the RAAS is overactivated to induce hypertension and accelerated DN. TTRhRen mice develop renin-dependent hypertension through constitutive expression of the human pro-renin cDNA, and when these mice are back-crossed to OVE26 mice, they develop significant albuminuria, mesangial expansion, tubulointerstitial fibrosis, and a decline in renal function by 20 weeks [48]. Similar results have been observed in diabetic mRen2 rats, which constitutively express murine renin cDNA [45]; however, this model is confounded by the development of malignant-phase hypertension [49]. This problem may be overcome by using Cyp1a1mRen2 rats in which the murine mRen2 cDNA is under the control of the Cyp1a1 promoter so that the timing and severity of hypertension may be controlled by adjusting the concentration of indole-3-carbinol in the diet [47•]. Concurrent induction of hyperglycaemia and renin-dependent hypertension in Cyp1a1mRen2 rats results in a 500-fold increase in albuminuria and moderate glomerulosclerosis and tubulointerstitial fibrosis, all features of moderately advanced human DN. However, none of these models exhibit all of the classical features of DN such as arteriolar hyalinosis.

## Monogenic Manipulations to Accelerate Nephropathy

To accelerate the development of nephropathy, researchers have employed mice in which specific genes have been targeted for knockout based on the known pathophysiology of human DN. For example, functional deficiency of endothelial nitric oxide synthase (eNOS) has been observed in patients with DN [50], and eNOS knockout (eNOS^−/−^) mice exhibit two of the key pathogenic mechanisms implicated in human DN: endothelial dysfunction and hypertension [51]. Induction of diabetes in eNOS^−/−^ mice by administration of streptozotocin [52] or by crossing to leptin-receptor deficient db/db mice [53] reproduces many features typical of human DN including the following: early onset albuminuria, decreased GFR, arteriolar hyalinosis, mesangial expansion, mesangiolysis, and nodular glomerulosclerosis. Importantly, these features are observed even in the nephropathy-resistant C57BL6/J strain [52]. However, only minimal tubulointerstitial fibrosis is observed in eNOS^−/−^ diabetic mice [52, 53].

Hypertriglyceridemia is common in patients with diabetes and is associated with the development of nephropathy [54]. Apolipoprotein E (ApoE) is implicated in clearance of triglycerides from the serum and ApoE deficient (ApoE^−/−^) mice develop marked hyperlipidemia and are widely used as a model of atherosclerosis [55]. Induction of diabetes with streptozotocin results in more severe renal injury in ApoE^−/−^ mice than wild-type controls, and this may in part due to hyperlipidemia, but also to accumulation of advanced glycation end-products (AGEs) [56]. The importance of AGEs in promoting nephropathy has also been demonstrated in mice that overexpress the receptor for advanced glycation end-products (RAGE) specifically in endothelial cells. When these mice were crossed with genetically diabetic mice, the RAGE-overexpressing mice develop more severe albuminuria and glomerulosclerosis compared with wild-type counterparts but do not develop tubulointerstitial disease or renal failure [57]. While therapies that target AGE were successful in these models, they have not as yet translated into clinical practice. These studies may provide a salutary lesson: when a novel therapy is tested in animals which have been genetically modified to promote overactivity of the target pathway, the therapy is very likely to be effective; however, this is not informative of the likely benefit in human disease.

## Use of Transcriptomic Profiling to Compare Pathways Activated in Human and Experimental DN

Our understanding of the molecular pathways activated in the kidneys of patients with DN have been aided by technological advances including the ability to separate the glomerular and tubulointerstitial compartments by laser capture microscopy, isolate RNA from archived formalin-fixed, paraffin-embedded tissue, and systematically assess gene expression using microarrays or RNA sequencing [58]. Much of this information has been made freely available to the nephrology community through Web-based interfaces such as Nephromine (www.nephromine.org). In parallel, advances in proteomics have enabled identification of specific peptides that are excreted in altered amounts in patients with DN [59] or a global urinary peptidomic signature that is characteristic of DN [60]. Researchers are now employing similar transcriptomic and peptidomic techniques to determine which animal models best replicate the molecular pathophysiology of human disease [8, 61••, 62].

The glomerular transcriptome in three murine models of DN (streptozotocin-induced diabetes on the DBA/2 background, db/db mice, and eNOS^−/−^ db/db mice) has been systematically compared with that from humans with type 2 diabetes and biopsy-proven early DN [61••]. The transcriptomic changes in the murine models typically resembled the pattern observed in patients with microalbuminuria rather than overt nephropathy. The human DN transcriptome was more similar to eNOS^−/−^ db/db mice than the other animal models tested, supporting biochemical and pathological data that suggest that the eNOS^−/−^ mouse may be a more representative model of human disease [52, 53]. For many pathways that are differentially expressed in the glomerulus in human DN, a similar pattern of expression was observed in a just one of murine models, implying that choice of model will depend on the specific pathway a researcher wishes to study.

In the Cyp1a1mRen2 rat model up to 50 % of differentially expressed genes in the tubulointerstitium in human DN were also dysregulated in the renal cortex of hyperglycemic and hypertensive rats [47•]. Importantly, the majority of the changes in gene expression were in the same direction in the rats as in humans, in contrast to the discordant patterns that emerged when the renal transcriptome in murine models was compared with that from patients with overt proteinuria.

**Fig. 1:**
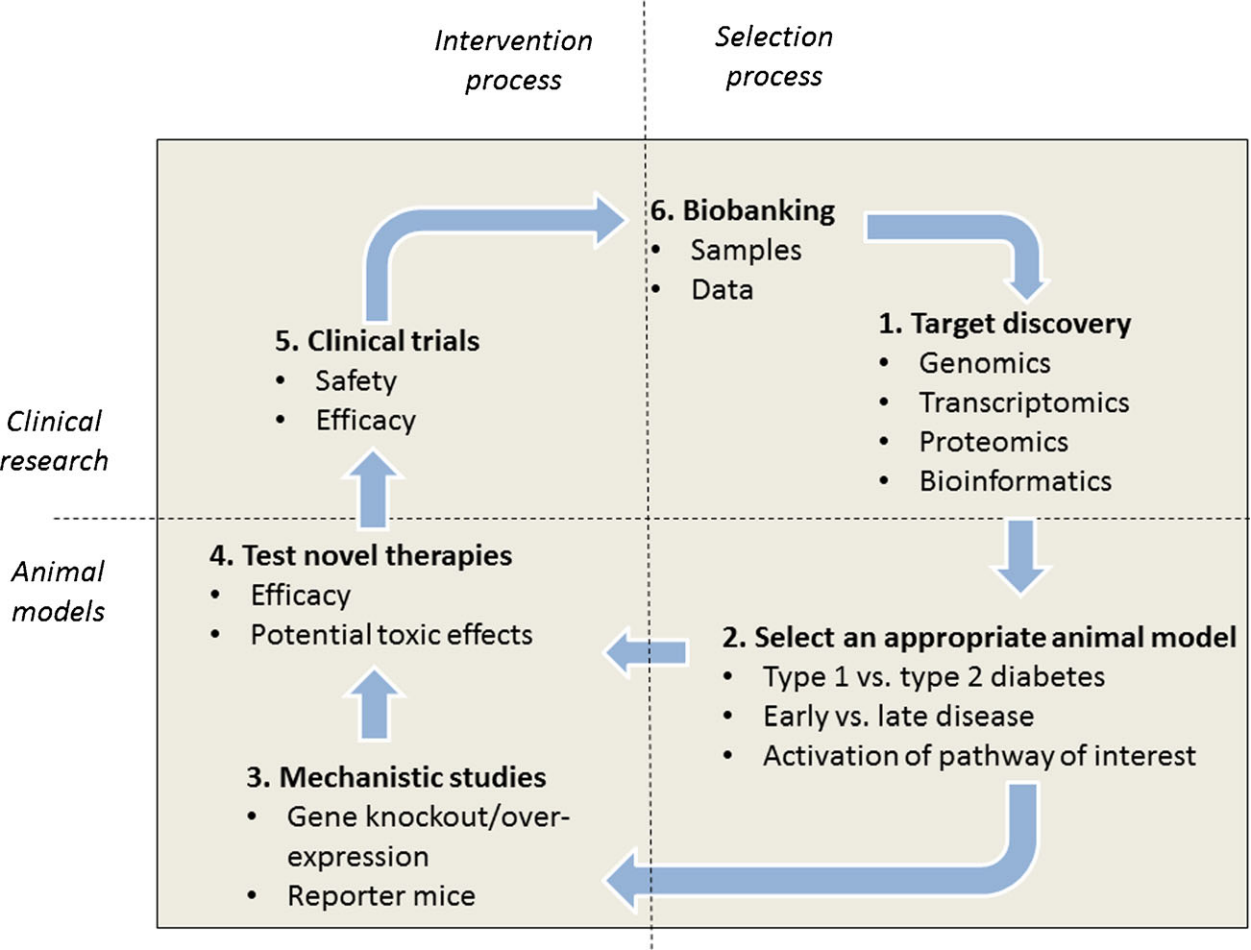
Schemata for discovery of novel therapeutic agents. *1*, High-throughput, nonbiased “-omic” approaches have identified hundreds of molecules that are associated with the development of human diabetic nephropathy. *2*, Whether these molecules could be targeted to slow progression of nephropathy may be determined using the most appropriate animal model for the specific research question. *3*, Genetically modified rodents may offer mechanistic insight and suggest whether development of a therapy is warranted. *4*, Pharmaceutical agents may be tested for efficacy and potential side effects. *5*, Therapies that are successful in robust animal studies may be taken forward into clinical trials. *6*, Samples and data from these trials may be “biobanked” to provide further mechanistic insight toward refining therapies

A major implication of the results from these -omic studies is that researchers must consider a number of issues in selecting the best animal model for their investigation (Fig. 1). Firstly, they must be clear regarding the relevant stage of human DN that they wish to replicate, with standard murine models reflecting the pathophysiology of early, but not late DN. Additional relevant injurious stimuli, such as hypertension, may be required to model progressive disease. Secondly, they may employ transcriptomic data to select the model which best recapitulates the activation status of their specific therapeutic target pathway. More rational selection of the most appropriate animal model may render research more efficient and improve the likelihood that the results will translate into clinical practice.

## Modeling Regression of DN

It is now recognized that DN does not always progress inexorably toward end-stage kidney disease, but that regression may also occur. Albuminuria may regress in up to 50 % of patients, particularly in those with optimal blood glucose and blood pressure control [63]. More remarkably, regression of established glomerulosclerosis and tubulointerstitial fibrosis has been observed in patients with moderately advanced DN who achieve sustained normoglycaemia after receiving a pancreas transplant [64, 65]. However, the pathways that promote regression remain poorly understood, in part because serial biopsies are rarely performed in patients who are responding to treatment.

To identify the pathways that promote regression, a number of rodent models have been employed. In BTBR ob/ob mice, administration of recombinant leptin for 8 weeks to reverse the genetic leptin deficiency promoted weight loss and improved glycemia control. This was accompanied by regression of albuminuria and glomerulosclerosis; however, there was no change in the severity of tubulointerstitial fibrosis [66••]. Importantly, leptin therapy, but not ACE inhibition, restored the mean number of podocytes in each glomerulus, implying that therapies other than renin-angiotensin system blockade are likely to be required to promote nephron regeneration in patients with DN.

In the Cyp1a1mRen2 rat, after 28 weeks of hyperglycaemia and hypertension, a reduction in albuminuria was observed following optimization of glycemic control by implanting insulin pellets subcutaneously and normalizing blood pressure through removal of indole-3-carbinol from the diet [67]. After 8 weeks of tight glycemic and blood pressure control, the expression of genes encoding extracellular matrix components reverted toward control levels, suggesting that tight control was sufficient to switch off new scar production. However, there was no change in the severity of glomerulosclerosis or tubulointerstitial fibrosis, implying that addition therapies may be required to accelerate degradation of established scar.

## Conclusion

In summary, animal models have been of limited utility in understanding the pathogenesis of DN, in part because no model exhibits all of the key features of human disease. Targeting additional genes for knockout either in isolation or in combination with known nephropathy susceptibility genes such as eNOS may refine existing models, although it is important to acknowledge that complete loss of gene expression is rarely observed in human disease. When reporting the phenotype of novel models, in addition to describing the functional and pathological findings, the transcriptomic changes in the kidney should be assessed for relevance to human disease. Going forward, it is likely that researchers will use transcriptomic data freely available on platforms such as Nephromine to select the animal model that best recapitulates the activation status of their pathway of interest in human disease. By rationalizing the selection of the most appropriate animal model for any given therapy, we may improve the likelihood that encouraging preclinical findings are successfully translated into clinical practice.
